# Relationship between the cumulative exposure to atherogenic index of plasma and ischemic stroke: a retrospective cohort study

**DOI:** 10.1186/s12933-023-02044-7

**Published:** 2023-11-15

**Authors:** Huancong Zheng, Kuangyi Wu, Weiqiang Wu, Guanzhi Chen, Zekai Chen, Zefeng Cai, Zhiwei Cai, Yulong Lan, Shouling Wu, Youren Chen

**Affiliations:** 1 Department of Cardiology, Second Affiliated Hospital of Shantou University Medical College, Shantou, China; 2 Shantou University Medical College, Shantou, China; 3 Cardiac Arrhythmia Center, Fuwai Hospital, National Center for Cardiovascular Diseases, Chinese Academy of Medical Sciences and Peking Union Medical College, Beijing, China; 4 Department of Epidemiology, University Medical Center Groningen, University of Groningen, Groningen, the Netherlands; 5 Centre for Precision Health, Edith Cowan University School of Medical and Health Sciences, Joondalup, Australia; 6 Department of Cardiology, Kailuan General Hospital, Tangshan, China

**Keywords:** Atherogenic index of plasma, Cumulative exposure, Ischemic Stroke

## Abstract

**Background:**

Atherogenic index of plasma (AIP) has been demonstrated as a surrogate marker for ischemic stroke, but there is limited evidence for the effect of long-term elevation of AIP on ischemic stroke. Therefore, we aimed to characterize the relationship between cumulative exposure to AIP and the risk of ischemic stroke.

**Methods:**

A total of 54,123 participants in the Kailuan Study who attended consecutive health examinations in 2006, 2008, and 2010 and had no history of ischemic stroke or cancer were included. The time-weighted cumulative AIP (cumAIP) was calculated as a weighted sum of the mean AIP values for each time interval and then normalized to the total duration of exposure (2006–2010). Participants were divided into four groups according to quartile of cumAIP: the Q1 group, ≤−0.50; Q2 group, − 0.50 to − 0.12; Q3 group, − 0.12 to 0.28; and Q4 group, ≥ 0.28. Cox proportional hazard models were used to evaluate the relationship between cumAIP and ischemic stroke by calculating hazard ratios (HRs) and 95% confidence intervals (95% CIs).

**Results:**

After a median follow-up of 11.03 years, a total of 2,742 new ischemic stroke events occurred. The risk of ischemic stroke increased with increasing quartile of cumAIP. After adjustment for potential confounders, Cox regression models showed that participants in the Q2, Q3, and Q4 groups had significantly higher risks of ischemic stroke than those in the Q1 group. The HRs (95% CIs) for ischemic stroke in the Q2, Q3, and Q4 groups were 1.17 (1.03, 1.32), 1.33 (1.18, 1.50), and 1.45 (1.28, 1.64), respectively. The longer duration of high AIP exposure was significantly associated with increased ischemic stroke risk.

**Conclusions:**

High cumulative AIP is associated with a higher risk of ischemic stroke, which implies that the long-term monitoring and maintenance of an appropriate AIP may help prevent such events.

**Supplementary Information:**

The online version contains supplementary material available at 10.1186/s12933-023-02044-7.

## Background

Stroke is a common disease globally and is associated with high mortality and high disability rates [1]. According to the 2019 Global Burden of Disease System analysis of data collected between 1990 and 2019, although the age-standardized incidences decreased significantly, the numbers of strokes and stroke-related deaths worldwide still increased significantly during this period [2]. Stroke can be divided into ischemic or hemorrhagic forms according to the nature of the underlying neurological pathology. Ischemic stroke is the most common subtype of pathological stroke, accounting for 85% of the total [3]. Therefore, it is essential to identify individuals at high risk of stroke, and especially ischemic stroke, and to implement effective preventive measures.

Previous studies have shown that atherosclerosis is the most common cause of ischemic stroke and that dyslipidemia is an important risk factor for the development of atherosclerosis [4, 5]. Dyslipidemia, including high circulating triglyceride (TG), total cholesterol (TC), low-density lipoprotein-cholesterol (LDL-C), and non-high-density lipoprotein-cholesterol (non-HDL-C) concentrations, increase the risk of ischemic stroke [6–9]. The atherogenic index of plasma (AIP) was first proposed as a biomarker of plasma atherosclerosis by Dobiásová and Frohlich, which is calculated as log (TG/HDL), and reflects the circulating concentrations of TG and HDL-C [10]. Recently, a growing number of studies have suggested that AIP may be a potential biomarker for atherosclerosis and CVD risk [11]. Previous studies have shown that high AIP is positively associated with the risk of CVD (e.g., myocardial infarction and ischemic stroke) [12–14], and patients with high AIP are more likely to experience cardiovascular events. However, most previous studies of the relationship between AIP and ischemic stroke have used a single measure, and few studies have been conducted to demonstrate long-term exposure to AIP and its effect on ischemic stroke risk. Furthermore, due to the short follow-up and limited sample size, the cumulative effect remains unclear. Therefore, in the present study, we used a large Kailuan study cohort with the aim of evaluating the relationship between the cumulative AIP (including AIP values and duration of exposure to high AIP) and ischemic stroke.

## Methods

### Study population

**Fig. 1:**
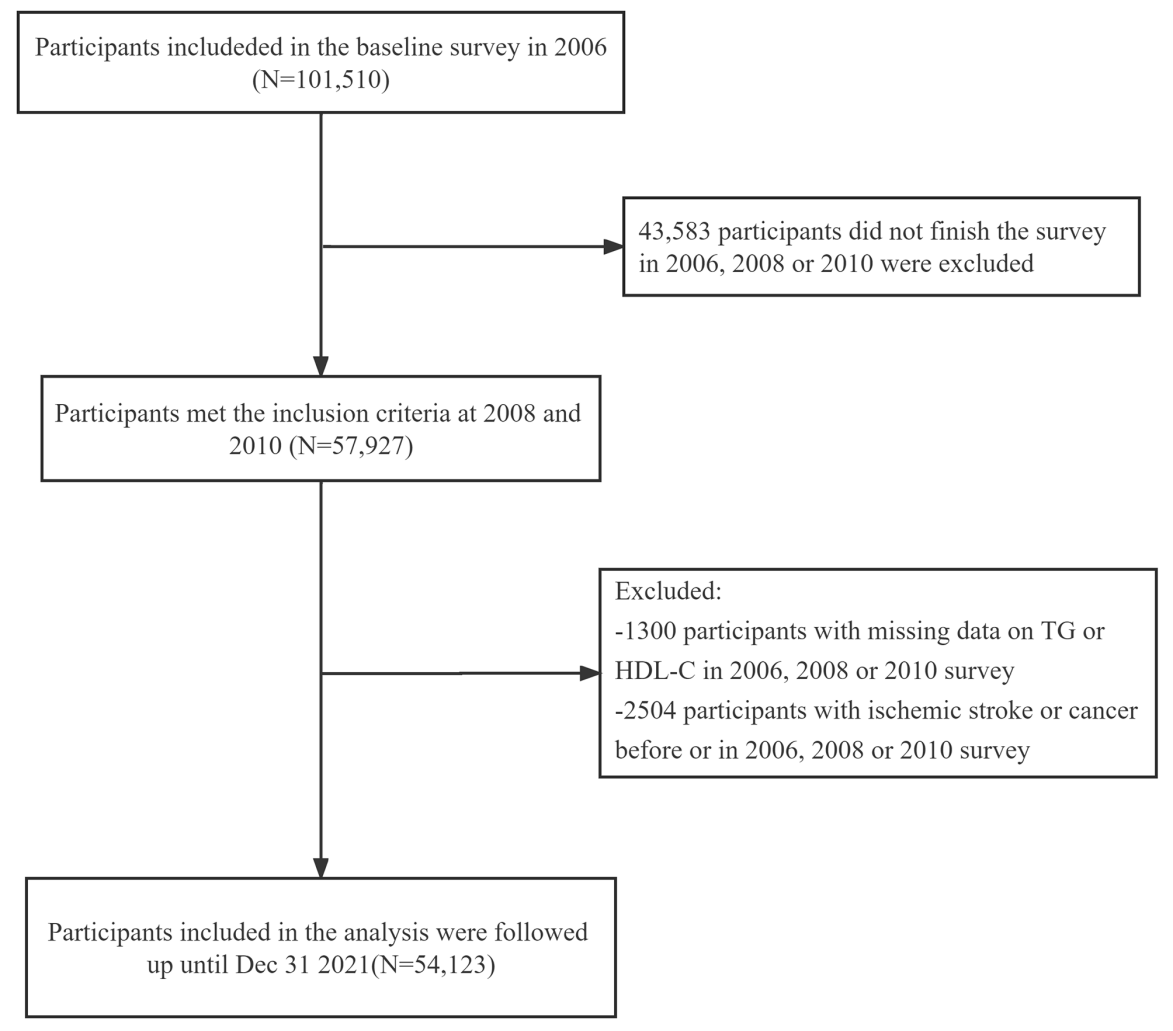
Flow chart for the inclusion of participants in the study

The Kailuan study is a large prospective cohort study of 101,510 participants (81,110 men and 20,400 women, aged 18–98 years) that commenced in 2006 in Tangshan, China. The participants are examined once every 2 years to identify the risk factors associated with, and assess the progression of, chronic non-communicable diseases. The detailed research methods have been described previously [15, 16]. Participants who met the following criteria were included in the present study: (1) those who all participated in the first three health examinations (2006–2007, 2008–2009 and 2010–2011); (2) those with a complete set of data for TG and HDL-C from three health examinations; and (3) those who agreed to participate in the study and gave their written informed consent. Participants with a history of ischemic stroke or cancer before the third health examination (2010–2011) were excluded, and a total of 54,123 participants were finally included (Fig. 1). The study conformed to the principles of the Declaration of Helsinki and was approved by the Ethics Committee of Kailuan General Hospital (approval number 200,605).

### Data collection and definitions

All the participants underwent baseline and follow-up examinations at one of the 11 hospitals in the Kailuan community, where questionnaires were completed under the supervision of specialized staff, to collect information regarding the age, sex, lifestyle-related factors (alcohol consumption, smoking status, and physical activity), medical history (cardiovascular disease, diabetes mellitus, or hypertension), and history of the use of medication (antihypertensive, lipid-lowering, or hypoglycemic). Blood pressure measurements were made at least twice by experienced physicians using a calibrated mercury sphygmomanometer that was placed on the right upper arm of participants. If the difference between the two measurements was ≥ 5 mmHg, the blood pressure of the participant was measured again, and the mean of the three blood pressure values was recorded. Height and body mass were measured under standardized conditions, according to a standardized protocol, to the nearest 0.1 cm and 0.1 kg, respectively. Body mass index (BMI) was calculated by dividing body mass (kg) by the square of height (m^2^). Current smokers were defined as those who had smoked a mean of ≥ 1 cigarette/day for > 1 year and were still smoking in the last year. Current drinkers were defined as those who had been drinking for ≥ 1 year, had a mean alcohol consumption of ≥ 100 ml/ day, and were still drinking in the last year. Active physical exercise was defined as taking exercise ≥ 3 times per week for at least 30 min on each occasion [17]. Diabetes was defined using a fasting plasma glucose (FPG) concentration of ≥ 7.0 mmol/L, and/or a clear history of diabetes, and/or the use of hypoglycemic drugs [18]. Hypertension was defined using a blood pressure ≥ 140/90 mmHg, and/or a clear history of hypertension, and/or the use of antihypertensive drugs [19].

### Measurement of biochemical indices and calculation of AIP

The participants were fasted for at least 8 h, then blood samples were collected from their elbow vein in the morning on the day of the physical examination, and serum samples were obtained by centrifugation and analyzed using a Hitachi 747 automatic analyzer (Tokyo, Japan) with respect to the TG, FPG, hs-CRP, HDL-C, LDL-C, TC, at the central laboratory of Kailuan Hospital. The serum creatinine concentration was determined using a creatine oxidase assay (Creatinine Kit, BioSino BioTechnology and Science Inc, Beijing, China) [20]. Glomerular filtration rate (eGFR) was calculated using the creatinine-based Chronic Kidney Disease Epidemiology Collaboration (CKD-EPI 2009) equation [21].

AIP was calculated as Log(TG/HDL-C) [10], and the time-weighted cumulative AIP (cumAIP) was calculated as the weighted sum of the mean AIP values for each time interval, which was then normalized to the total duration of exposure [22] (between 2006 and 2010). The time-weighted cumAIP was calculated as: [(AIP_2006_ + AIP_2008_)/2 × time _2006–2008_ + (AIP_2008_ + AIP_2010_)/2 × time_2008–2010_)]/time_2006–2010_; where AIP_2006_, AIP_2008_, and AIP_2010_ are the AIP indexes for the first, second, and third examinations, respectively; time_2006–2008_ and time_2008–2010_ are the periods of time between consecutive examinations intervals (years); and the time from 2006 to 2010 shows the time interval between the first and the third visit. The participants were then placed into group Q1 ( ≤ − 0.50), group Q2 (− 0.50 to − 0.12), group Q3 (− 0.12 to 0.28), or group Q4 (≥ 0.28) according to quartiles of the cumAIP.

Previous studies have shown that participants with a high AIP are at a higher risk of ischemic stroke [13], and in the present study, a high AIP exposure was defined as being one in the highest quartile of AIP [23]. The duration of exposure to a high AIP was defined as the period of time during which the AIP of a participant was high over the period of the three examinations, and was quantified as 0 years (never had a high AIP), 2 years (had a high AIP once), 4 years (had a high AIP twice), or 6 years (had a high AIP at all three examinations).

### Assessment of ischemic Stroke

The present study commenced at the 2010 annual health check and the end point of follow-up was the occurrence of a first ischemic stroke. The incidence of ischemic stroke was assessed annually during the follow-up period. Potential ischemic stroke events were identified using social health insurance records, hospital discharge summaries, death certificates, and questionnaires, and potential ischemic stroke cases included cases identified on the basis of the first 3 sources identified through ICD-10th revision code 163.x or self-reported cases in questionnaires [24]. According to World Health Organization criteria, ischemic stroke was diagnosed on the basis of neurologic signs, clinical symptoms, and neuroimaging, including computed tomography and magnetic resonance imaging [25–27], neuroimaging was performed in all cases of ischemic stroke in this study. In addition, a group of experienced experts collects and reviews annual hospital discharge records to identify suspected cases of ischemic stroke. Fatal cases of ischemic stroke were identified from medical records, autopsy reports, or death certificates with ischemic stroke as the underlying cause of death. Cases of nonfatal ischemic stroke were classified as sudden onset focal neurological deficits with a vascular mechanism lasting more than 24 h. All ischemic stroke outcomes were validated by the Data Safety Monitoring Board and the Arbitration Committee for Clinical Outcomes. The first ischemic stroke was used as the endpoint for those who had two or more ischemic stroke events, and the end of follow-up for those who did not have an ischemic stroke event was the time of their death or final follow-up examination (December 31, 2021).

### Statistical analysis

Statistical analyses were performed using SAS v.9.4 (SAS Institute, Inc, Cary, NC, USA). Continuous normally distributed variables are expressed as mean ± standard deviation (SD) and continuous skewed variables are expressed as median and interquartile range. Comparisons of continuous variables were performed using the Kruskal–Wallis test or one-way ANOVA, and chi-square tests were used for categorical variables. Missing covariate data were imputed using multiple imputation for chained equations. The Kaplan–Meier method was used to calculate the prevalence of ischemic stroke for each quartile group, and the log-rank test was used for comparisons. In addition, two Cox proportional risk models were constructed to assess the relationships of cumAIP and the duration of exposure to high AIP with the risk of ischemic stroke by calculating hazard ratios (HRs) and 95% confidence intervals (95% CIs). Model 1 was adjusted for sex and age. Model 2 was further adjusted for BMI, LDL-C, TC, hs-CRP, eGFR, educational level, current smoking status, current drinking status, and the presence of hypertension or diabetes. Model 3 was further adjusted for the use of antihypertensive, hypoglycemic, and/or lipid-lowering drugs. Subgroup analyses were performed on the basis of age (< 65 years vs. ≥65 years), sex (male vs. female), BMI (< 28 kg/m^2^vs. ≥28 kg/m^2^), and hypertension (yes vs. no). In addition, to assess the relationship between long-term exposure to the AIP and the risk of developing ischemic stroke, we used the C-statistic, integrated discriminant improvement (IDI), and net reclassification index (NRI) to predict the risk of ischemic stroke, based on the conventional China-par model [28].

We performed several sensitivity analyses to evaluate the robustness of the results. First, because a change in the cumAIP may be a consequence of an impending ischemic stroke (i.e., reverse causality), we performed a lagged analysis that excluded participants who experienced an ischemic stroke event within the first year. In addition, considering the possible impact of atrial fibrillation or coronary artery disease on ischemic stroke, we excluded participants with history of atrial fibrillation or coronary artery disease. Third, to explore whether the relationship between cumAIP and ischemic stroke was confounded by the use of medication, we excluded participants who were taking anti-hypertensive, lipid-lowering, and/or hypoglycemic drugs. Finally, cumulative AIP was calculated using different methods or additional adjustments were made for baseline AIP. A two-sided *p* < 0.05 was regarded as indicating statistical significance.

## Results

### Baseline characteristics

A total of 57,927 participants were enrolled on the basis of their attendance at all three physical examinations, in 2006, 2008, and 2010. Of these, 2,504 had a history of ischemic stroke or cancer at the time of the 2006 physical examination, and those who had experienced ischemic stroke or cancer during the 2006–2010 period were excluded, as were 1,300 with missing TG or HDL-C data. Therefore, data for 54,123 participants were analyzed. The baseline characteristics of the participants were shown in Table 1. The mean age of the participants was 49.05 ± 11.84 years, and they comprised 41,303 (76.3%) men and 12,820 (23.7%) women. The participants were divided into Q1–Q4 groups on the basis of the cumAIP quartile, and when compared with the lowest quartile (Q1) group, the participants with higher cumAIP were more likely to be male; to have higher BMI, blood pressure, FPG, TG, hs-CRP, TC, and TG; to have lower HDL-C and eGFR; to be current smokers and drinkers; to have diabetes mellitus and hypertension; and to be taking antihypertensive, lipid-lowering, and hypoglycemic drugs.

**Table 1:** Baseline characteristics of participants by cumulative AIP quartile

Variables	Total	Q1(≤-0.50)	Q2(-0.50-0.12)	Q3(-0.12-0.28)	Q4(≥ 0.28)	P
N	54,123	13,530	13,531	13,531	13,531	-
Age, years	49.05 ± 11.84	49.35 ± 12.41	49.38 ± 12.11	49.08 ± 11.71	48.41 ± 11.05	< 0.01
Male, N(%)	41,303(76.3)	9269.0(68.5)	10,047(74.3)	10,721(79.2)	11,266(83.3)	< 0.01
SBP, mmHg	129.39 ± 16.83	125.33 ± 17.16	128.67 ± 16.87	130.62 ± 16.19	132.94 ± 16.14	< 0.01
DBP, mmHg	83.52 ± 9.20	80.75 ± 9.08	82.89 ± 9.07	84.36 ± 8.76	86.07 ± 9.02	< 0.01
BMI, kg/m^2^	25.02 ± 3.16	23.38 ± 2.89	24.66 ± 2.99	25.53 ± 2.96	26.51 ± 2.96	< 0.01
HDL-C, mmol/L	1.54 ± 0.32	1.76 ± 0.36	1.57 ± 0.27	1.47 ± 0.24	1.36 ± 0.26	< 0.01
LDL-C, mmol/L	2.50 ± 0.64	2.34 ± 0.65	2.54 ± 0.62	2.59 ± 0.60	2.52 ± 0.64	< 0.01
FPG, mmol/L	5.54 ± 1.38	5.26 ± 1.12	5.46 ± 1.26	5.60 ± 1.38	5.86 ± 1.65	< 0.01
eGFR, ml/min/1.73m^2^	87.41 ± 17.55	89.62 ± 16.97	87.33 ± 17.48	85.66 ± 17.56	87.03 ± 17.94	< 0.01
TC, mmol/L	4.97 ± 0.85	4.82 ± 0.81	4.93 ± 0.81	4.99 ± 0.84	5.14 ± 0.92	< 0.01
TG,mmol/L	1.67 ± 1.15	0.82 ± 0.22	1.20 ± 0.27	1.63 ± 0.40	3.02 ± 1.48	< 0.01
hs-crp, mg/L	2.59 ± 3.60	2.38 ± 3.71	2.59 ± 3.76	2.56 ± 3.51	2.83 ± 3.40	< 0.01
AIP index_2006_	-0.10 ± 0.68	-0.75 ± 0.42	-0.31 ± 0.39	0.05 ± 0.44	0.60 ± 0.57	< 0.01
AIP index_2008_	-0.08 ± 0.69	-0.86 ± 0.35	-0.30 ± 0.28	0.07 ± 0.28	0.76 ± 0.53	< 0.01
AIP index_2010_	-0.10 ± 0.73	-0.81 ± 0.46	-0.29 ± 0.41	0.05 ± 0.43	0.67 ± 0.63	< 0.01
cumAIP	-0.09 ± 0.60	-0.82 ± 0.24	-0.30 ± 0.11	0.06 ± 0.11	0.70 ± 0.38	< 0.01
Current smoker, N(%)	20,735(38.3)	4382.0(32.4)	4877.0(36.0)	5272.0(39.0)	6204.0(45.9)	< 0.01
Current drinker, N(%)	19,200(35.5)	4283.0(31.7)	4403.0(32.5)	4738.0(35.0)	5776.0(42.7)	< 0.01
Physical activity, N(%)	7764.0(14.3)	1996.0(14.8)	1993.0(14.7)	1904.0(14.1)	1871.0(13.8)	0.07
High school or above, N(%)	15,076(27.9)	3595.0(26.6)	3720.0(27.5)	3826.0(28.3)	3935.0(29.1)	< 0.01
Hypertension, N(%)	25,861(47.8)	5035.0(37.2)	6081.0(44.9)	6889.0(50.9)	7856.0(58.1)	< 0.01
Diabetes mellitus, N(%)	6012.0(11.1)	814.00(6.02)	1190.0(8.79)	1595.0(11.8)	2413.0(17.8)	< 0.01
Hypoglycemic drugs, N(%)	3123.0(5.77)	459.00(3.39)	657.00(4.86)	822.00(6.07)	1185.0(8.76)	< 0.01
Anti-hypertensive drugs, N(%)	8395.0(15.5)	1358.0(10.0)	1837.0(13.6)	2368.0(17.5)	2832.0(20.9)	< 0.01
Lipid-lowering drugs, N(%)	1481.0(2.74)	158.00(1.17)	278.00(2.05)	358.00(2.65)	687.00(5.08)	< 0.01

AIP atherogenic index of plasma, SBP systolic blood pressure, DBP diastolic blood pressure, HDL-C high-density lipoprotein cholesterol, LDL-C low-density lipoprotein cholesterol, TG triglyceride, FPG fasting plasma glucose, hs-CRP high-sensitivity C reactive protein, eGFR estimated glomerular filtration rate

### Relationship between cumAIP and the risk of ischemic stroke

**Fig. 2:**
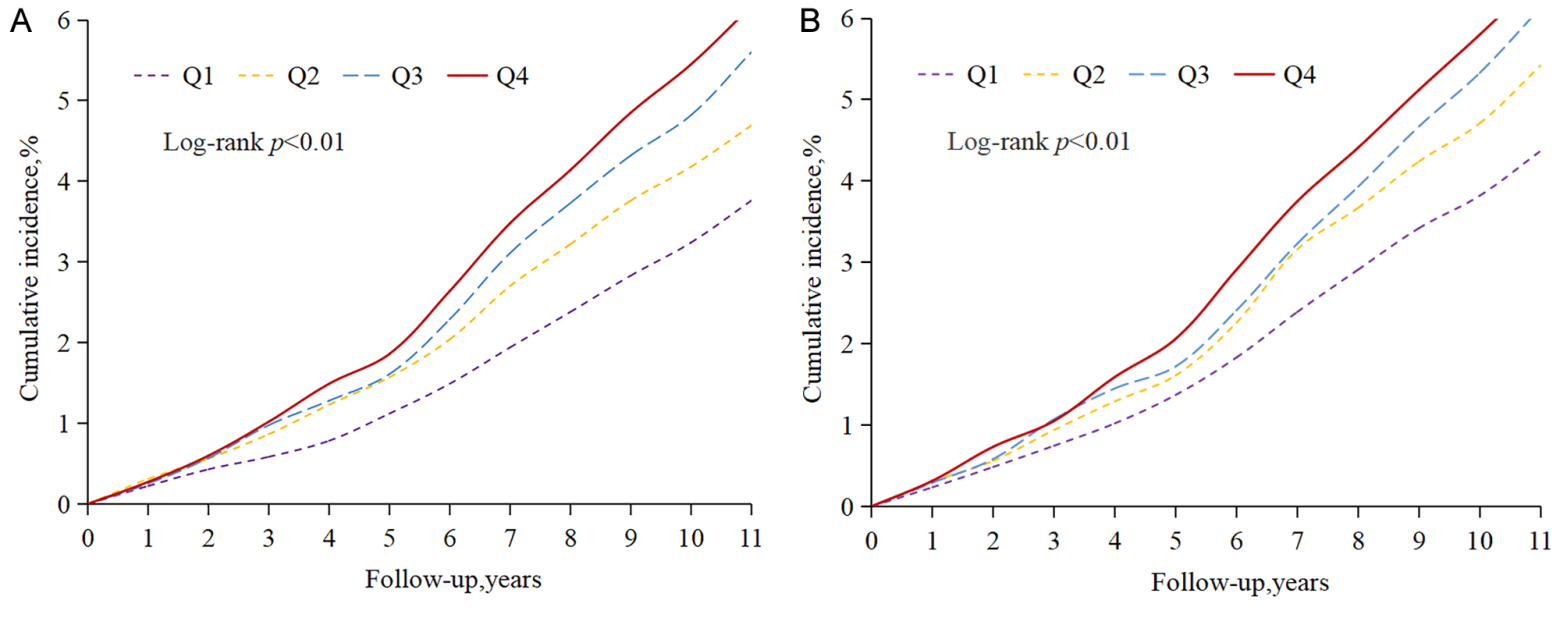
Kaplan–Meier incidence rate of ischemic stroke by AIP. **A** Quartiles of cumulative AIP. **B** Exposure duration with a higher AIP

A total of 2,742 ischemic stroke events occurred during a median follow-up period of 11.03 years (interquartile range 10.67–11.32). The incident rate of ischemic stroke events in the Q1, Q2, Q3, and Q4 groups was 3.50, 4.38, 5.17, and 5.80 per 1,000 person-years, respectively. According to the Kaplan–Meier curves, the prevalence of ischemic stroke was highest among participants in the Q4 group and increased from the Q1 to the Q4 group (log-rank test *P* < 0.01; Fig. 2A). After adjustment for potential confounding factors, compared with those of the Q1 group, the HRs and 95% CIs of the Q2–Q4 groups in Model 3 were 1.17 (1.03, 1.32), 1.33 (1.18, 1.50), and 1.45 (1.28, 1.64), respectively (Table 2).

**Table 2:** Association of cumulative AIP with ischemic stroke

	Case/Total	Incidence rate, per 1000 person-years	Model 1	Model 2	Model 3
Quartiles					
Q1	513/13,530	3.50	1.00	1.00	1.00
Q2	638/13,531	4.38	1.25(1.12,1.41)	1.17(1.04,1.32)	1.17(1.03,1.32)
Q3	752/13,531	5.17	1.50(1.34,1.68)	1.34(1.19,1.51)	1.33(1.18,1.50)
Q4	839/13,531	5.80	1.74(1.56,1.95)	1.47(1.30,1.66)	1.45(1.28,1.64)
P for trend			< 0.0001	< 0.0001	< 0.0001
Time exposure duration					
0 year	1297/29,549	4.07	1.00	1.00	1.00
2 years	630/11,613	5.04	1.29(1.17,1.42)	1.18(1.07,1.30)	1.17(1.06,1.30)
4 years	442/7222	5.71	1.47(1.32,1.64)	1.30(1.16,1.45)	1.28(1.14,1.43)
6 years	373/5739	6.09	1.58(1.41,1.77)	1.33(1.17,1.50)	1.31(1.15,1.48)
P for trend			< 0.0001	< 0.0001	< 0.0001

Model 1: adjust for age, sex

Model 2: included variables in model 1 and further BMI, LDL-C, TC, hs-CRP, eGFR, current smoker, current drinker, physical activity, education level, hypertension, diabetes mellitus

Model 3: included variables in model 2 and further hypoglycemic drugs, anti-hypertensive drugs and lipid-lowering drugs

### Relationship between the duration of exposure to a high AIP and the incidence of ischemic stroke

Table 2 shows that the cumulative incidence of ischemic stroke gradually increased with the duration of exposure to high AIP (*P* < 0.01; Fig. 2B). The incident rate of ischemic stroke events was 4.07, 5.04, 5.71 and 6.09 per 1,000 person-years in the 0-, 2-, 4- and 6-year groups, respectively (log-rank test, two-sided *p* < 0.05). After adjustment for potential confounding factors, compared with those of the unexposed group (0 years of exposure), the risks of the 2-, 4-, and 6-year exposure groups (Model 3) were 1.17 (1.06, 1.30), 1.28 (1.14, 1.43) and 1.31 (1.15, 1.48) higher, respectively (Table 2).

### Results of the subgroup and sensitivity analyses

The results of subgroup analyses are shown in Table 3. We stratified the cumAIP by age, sex, BMI, and the presence of hypertension, and found no significant interactions between the cumAIP and these parameters. The exclusion of participants who experienced outcome events that occurred in the first year of follow-up or of those with a history of atrial fibrillation or coronary artery disease generated results that were consistent with those of the primary analysis (Additional file 1, Tables S1, S2 and S3). Furthermore, the results remained stable after the exclusion of participants who were taking antihypertensive, lipid-lowering, or hypoglycemic drugs (Additional file 1, Tables S4). Finally, there was no substantial change in the association between cumulative AIP and ischemic stroke risk after calculating cumulative AIP using different methods or additional correction for baseline AIP (Additional file 1, Tables S5 and S6).

**Table 3:** Subgroup analyses for the association of cumulative AIP with ischemic stroke

	Age		Sex		Bmi		Hypertension	
	< 65 years	≥ 65years	Male	Female	< 28 kg/m^2^	≥ 28 kg/m^2^	Yes	No
Quartiles								
Q1	1.00	1.00	1.00	1.00	1.00	1.00	1.00	1.00
Q2	1.11(0.97,1.27)	1.31(1.01,1.71)	1.15(1.01,1.32)	1.17(0.84,1.61)	1.20(1.05,1.36)	1.04(0.74,1.45)	1.16(0.99,1.35)	1.13(0.91,1.40)
Q3	1.24(1.09,1.42)	1.43(1.09,1.88)	1.31(1.15,1.49)	1.39(1.01,1.91)	1.34(1.18,1.52)	1.35(0.99,1.84)	1.26(1.09,1.46)	1.44(1.17,1.78)
Q4	1.30(1.13,1.48)	1.56(1.16,2.10)	1.43(1.26,1.64)	1.38(0.99,1.91)	1.55(1.36,1.77)	1.29(0.95,1.75)	1.33(1.15,1.54)	1.57(1.26,1.95)
P for interaction	0.7840		0.7018		0.2057		0.0722	
Time exposure duration								
0 year	1.00	1.00	1.00	1.00	1.00	1.00	1.00	1.00
2 years	1.10(0.99,1.23)	1.29(1.02,1.64)	1.16(1.04,1.29)	1.23(0.94,1.61)	1.18(1.06,1.32)	1.17(0.95,1.46)	1.08(0.95,1.)	1.32(1.09,1.58)
4 years	1.19(1.05,1.45)	1.38(1.03,1.85)	1.30(1.15,1.47)	1.09(0.78,1.52)	1.36(1.19,1.55)	1.18(0.94,1.48)	1.17(1.02,1.34)	1.38(1.10,1.72)
6 years	1.21(1.06,1.39)	1.18(0.82,1.71)	1.23(1.08,1.42)	1.65(1.19,2.27)	1.40(1.21,1.63)	1.23(0.97,1.55)	1.17(1.01,1.36)	1.40(1.09,1.80)
P for interaction	0.5981		0.1898		0.0971		0.1985	

Model adjusted for age, sex, BMI, LDL-C, TC, hs-CRP, eGFR, current smoker, current drinker, physical activity, education level, hypertension, diabetes mellitus, hypoglycemic drugs, anti-hypertensive drugs and lipid-lowering drugs

### Incremental predictive value of cumAIP

We separately added the cumAIP and the AIP in 2006 and 2010 to the conventional China-PAR prediction model, and found that the cumAIP had the highest predictive value for ischemic stroke, with a C-index of 71.02%. When the cumAIP prediction model was compared with the original China-PAR prediction model (C-index was 70.58%), the 2006 prediction model (C-index was 70.61%) and the 2010 prediction model (C-index was 70.74%), the C-index of the cumAIP prediction model increased by 0.44%, 0.41%, and 0.28%, the IDI increased by 0.0411%, 0.0259%, and 0.0053%, and the continuous NRI increased by 13.46%, 2.40%, and 0.67%, respectively (Additional file 1, Table S7).

## Discussion

In this prospective cohort study of 54,123 participants in the Kailuan study, we found that high cumAIP is a risk factor for new-onset ischemic stroke. High cumAIP increases the risk of new-onset ischemic stroke, independently of conventional risk factors. In addition, we found that long-term exposure to a high AIP increases the risk of ischemic stroke. Cumulative AIP may be more predictive of ischemic stroke risk compared with single AIP.

We found that the cumulative exposure to a high AIP was an independent risk factor for new-onset ischemic stroke. Similar findings have been made in previous studies regarding the relationship between AIP and the risk of ischemic stroke. Liu et al. [13] conducted a cohort study of 1,463 patients that had been admitted with acute ischemic stroke, and found that higher AIP was more strongly associated with adverse ischemic stroke outcomes compared with the lowest AIP quartile group. A statistical analysis of 10,2009 participants in the National Health Insurance-National Health Examination Cohort (NHIS-HEALS) showed that a high AIP increases the risk of cardiovascular disease [29]. In addition, a high AIP has been shown to be an independent risk factor for cardiovascular disease in older non-diabetic patients with hypertension [30]. However, they only examined AIP measured at a single time point and did not take into account changes in AIP over time, which would lead to potential regression dilution bias and could affect the accuracy of the results. In the present study, we found that participants with a high cumAIP were at a higher risk of ischemic stroke, and that those in the highest quartile were at the highest risk, with a multivariate-adjusted HR (95% CI) of 1.45 (1.28, 1.64), which suggests that cumulative exposure to a high AIP may be associated with a higher risk of ischemic stroke than a single exposure. In addition, we found a 31% higher risk of ischemic stroke with 6 years of exposure to a high AIP compared with the unexposed group. The risk of ischemic stroke increased with the degree of cumulative exposure to a high AIP.

Another important finding of this study is that cumulative AIP may be a better predictor of ischemic stroke risk than single AIP. Previous cohort studies have shown that cumulative exposure to risk factors such as AIP, blood pressure, lipid profile, and uric acid has a better impact on adverse outcomes such as cardiovascular disease than a single exposure [22, 31–33], which is consistent with the present findings. Based on the traditional China-Par model, we developed a risk prediction model for ischemic stroke by adding the AIP_2006_, AIP_2010_ and cumAIP, respectively. The results showed that the C-index (71.02%) of the cumAIP model was the highest, which was 0.41% and 0.28% higher than that of AIP_2006_(70.61%) and AIP_2010_(70.74%), respectively. The prediction ability of the model was improved after adding cumAIP. It is suggested that cumAIP may be a better predictor for ischemic stroke than single-measure AIP and may be more powerful in predicting ischemic stroke risk. However, this association still needs to be further validated by other large cohort studies.

To the best of our knowledge, few cohort studies have explored the AIP through repeated measures analysis. A previous Kailuan study showed that both cumAIP and hs-CRP are independently associated with a higher risk of incident type 2 diabetes (T2DM) [34]. In the Chinese Longitudinal Study of Health and Retirement of 8,760 participants, the differences in AIP between baseline and follow-up examinations were used to predict the risk of T2DM, and the results showed that, compared with people with maintained-low AIP, those who had maintained-high AIP or showed transitions from high-to-low AIP, or from low-to-high AIP, are at approximately 1.5 times higher risk of T2DM. However, the risk of T2DM did not decrease in the high-to-low AIP group as compared to the maintained-high AIP group [35]. However, the relationship between cumAIP and ischemic stroke has not been studied previously. The present findings suggest that cumulative exposure to high AIP and long-term exposure to high AIP are associated with a high risk of ischemic stroke, which highlights the importance of early detection and control of lipids in clinical practice.

The mechanism underlying the relationship between the cumulative exposure to AIP and the risk of ischemic stroke is unclear, but this may be related to atherosclerosis. First, AIP is a substitute for sdLDL particles and is negatively correlated with LDL-C particle size [10, 40]. An increase in AIP indicates a reduction in LDL particle diameter and an increase in the proportion of sdLDL, which promotes the development of atherosclerotic plaque [12]. AIP plays an important role in regulating the reverse cholesterol transport process, which is associated with the recycling or disposal of excess cholesterol. Elevated AIP may mean that adipocytes store excess TG as fat, which increases the accumulation of cholesterol crystals in the inner layers of atherosclerotic arteries, causing lumen narrowing and blockage, and ultimately leading to atherosclerosis formation [36, 37]. Second, atherosclerosis increases the risk of many chronic metabolic diseases, including hypertension, diabetes mellitus, and metabolic syndrome, which are important risk factors for ischemic stroke [38, 39], and a combination of these may further increase the risk of ischemic stroke. In addition, atherosclerosis is associated with platelet adhesion, activation, and aggregation, which may lead to hemodynamic disturbances, and therefore cerebral artery occlusion [40, 41]. Finally, participants with a long-term high AIP level were found to have higher BMI, SBP, DBP, and TC; and tended to be current smokers or current drinkers, all of which are common risk factors for ischemic stroke. Thus, it may lead to a significantly increased risk of ischemic stroke.

Our study has important implications for the prevention of ischemic stroke. The assessment of cumulative exposure to the AIP may help identify individuals who are at high risk of experiencing ischemic stroke in large long-term cohort studies. For the general population, the maintenance of appropriate TG and HDL-C concentrations and the control of cumAIP are important for the management of chronic disease. The strengths of the present study are that it is a large prospective cohort study with a long follow-up period that involved repeated measures of multiple laboratory variables. However, there are also some limitations. First, we adjusted for many potential confounders, but unmeasured or residual confounders may have affected the results. Second, the information regarding the lifestyle (physical activity, smoking status, and alcohol consumption) and medication (antihypertensive, hypoglycemic, and lipid-lowering drugs) of the participants was self-reported, which rendered it subject to recall bias. In addition, most of the participants were male coal miners living in northern China. Therefore, women were underrepresented, and the findings may not be directly generalizable to other populations. Last but not least, the lack of information on ischemic stroke subtypes (e.g., large artery atherosclerosis, small vessel occlusion, cardioembolism, etc.) in our Kailuan study cohort limited our ability to further explore the mechanistic link between AIP and ischemic stroke, and more studies are needed to validate our results in the future.

## Conclusions

In summary, we have shown that cumulative exposure to a high AIP is associated with a higher risk of ischemic stroke. Long-term exposure to a high AIP may increase the risk of ischemic stroke. These results emphasize that long-term monitoring and maintenance of appropriate AIP levels may help prevent ischemic stroke.

### Electronic supplementary material

Below is the link to the electronic supplementary material.

Supplementary Material 1

## Data Availability

The datasets used and/or analyzed during the present study are available from the corresponding author on reasonable request.

## Abbreviations

AIP: Atherogenic index of plasma
TG: Triglyceride
FPG: Fasting plasma glucose
TC: Total cholesterol
SBP: Systolic blood pressure
DBP: Diastolic blood pressure
hs-CRP: High-sensitivity C-reactive protein
BMI: Body mass index
HDL-C: High-density lipoprotein-cholesterol
LDL-C: Low-density lipoprotein-cholesterol
eGFR: estimated glomerular filtration rate
HR: Hazard ratio
CI: Confidence interval
