# Supplementary material for: Relationship between the cumulative exposure to atherogenic index of plasma and ischemic stroke: a retrospective cohort study
Source: Cardiovasc Diabetol. 2023 Nov 15;22:313. doi: 10.1186/s12933-023-02044-7 (PMC10652447; doi:10.1186/s12933-023-02044-7)
Supplement: Supplementary file 1 — Supplementary Material 1 [file 12933_2023_2044_MOESM1_ESM.docx]

**Additional file 1**

**Additional tables**

**Table S1** Sensitivity analysis of excluding outcome events within the first year of follow-up

**Table S2** Sensitivity analysis of excluding participants with the history of atrial fibrillation

**Table S3** Sensitivity analysis of excluding participants with the history of coronary artery disease

**Table S4** Sensitivity analysis of excluding participants with the use of anti-hypertensive drugs, hypoglycemic drugs

or lipid-lowering drugs

**Table S5** Sensitivity analysis of additionally adjusted for AIP index at baseline

**Table S6** Sensitivity analysis of using another method to calculate the cumulative AIP

**Table S7** Reclassifcation and discrimination statistics for cumulative AIP

|  | Case/Total | IR | HR(95%CI) |
| --- | --- | --- | --- |
| Quartiles |  |  |  |
| Q1 | 252/13500 | 1.89 | 1.00 |
| Q2 | 322/13489 | 2.42 | 1.15(1.01,1.30) |
| Q3 | 342/13497 | 2.57 | 1.34(1.19,1.52) |
| Q4 | 406/13495 | 3.07 | 1.46(1.29,1.66) |
| P for trend |  |  | <0.0001 |
| Time exposure duration |  |  |  |
| 0 year | 1228/29480 | 3.85 | 1.00 |
| 2 years | 595/11578 | 4.76 | 1.17(1.05,1.29) |
| 4 years | 422/7202 | 5.45 | 1.29(1.15,1.45) |
| 6 years | 355/5721 | 5.80 | 1.30(1.14,1.48) |
| P for trend |  |  | <0.0001 |

**Table S1** Sensitivity analysis of excluding outcome events within the first year of follow-up(n=142)

Note: IR, incidence rate per 1000-person years; Model adjusted for age, sex, BMI, LDL-C, TC, hs-CRP, eGFR, current smoker, current drinker, physical activity, education level, hypertension, diabetes mellitus, hypoglycemic drugs, anti-hypertensive drugs and lipid-lowering drugs.

|  | Case/Total | IR | HR(95%CI) |
| --- | --- | --- | --- |
| Quartiles |  |  |  |
| Q1 | 497/13330 | 3.44 | 1.00 |
| Q2 | 622/13340 | 4.33 | 1.16(1.03,1.32) |
| Q3 | 736/13363 | 5.13 | 1.33(1.18,1.51) |
| Q4 | 823/13356 | 5.76 | 1.44(1.28,1.63) |
| P for trend |  |  | <0.0001 |
| Time exposure duration |  |  |  |
| 0 year | 1263/29114 | 4.02 | 1.00 |
| 2 years | 614/11478 | 4.97 | 1.18(1.06,1.30) |
| 4 years | 436/7122 | 5.71 | 1.30(1.16,1.46) |
| 6 years | 365/5675 | 6.02 | 1.30(1.15,1.48) |
| P for trend |  |  | <0.0001 |

**Table S2** Sensitivity analysis of excluding participants with the history of atrial fibrilltion (n=734)

Note: IR, incidence rate per 1000-person years; Model adjusted for age, sex, BMI, LDL-C, TC, hs-CRP, eGFR, current smoker, current drinker, physical activity, education level, hypertension, diabetes mellitus, hypoglycemic drugs, anti-hypertensive drugs and lipid-lowering drugs.

.

|  | Case/Total | IR | HR(95%CI) |
| --- | --- | --- | --- |
| Quartiles |  |  |  |
| Q1 | 504/13387 | 3.49 | 1.00 |
| Q2 | 628/13340 | 4.37 | 1.17(1.04,1.33) |
| Q3 | 741/13273 | 5.20 | 1.36(1.20,1.53) |
| Q4 | 826/13207 | 5.84 | 1.49(1.31,1.69) |
| P for trend |  |  | <0.0001 |
| Time exposure duration |  |  |  |
| 0 year | 1278/29164 | 4.06 | 1.00 |
| 2 years | 618/11403 | 5.04 | 1.19(1.07,1.31) |
| 4 years | 435/7046 | 5.76 | 1.32(1.17,1.48) |
| 6 years | 368/5594 | 6.17 | 1.34(1.18,1.52) |
| P for trend |  |  | <0.0001 |

**Table S3** Sensitivity analysis of excluding participants with the history of coronary artery disease(n=916)

Note: IR, incidence rate per 1000-person years; Model adjusted for age, sex, BMI, LDL-C, TC, hs-CRP, eGFR, current smoker, current drinker, physical activity, education level, hypertension, diabetes mellitus, hypoglycemic drugs, anti-hypertensive drugs and lipid-lowering drugs.

**Table S4** Sensitivity analysis of excluding participants with the use of anti-hypertensive drugs, hypoglycemic drugs

|  | Case/Total | IR | HR(95%CI) |
| --- | --- | --- | --- |
| Quartiles |  |  |  |
| Q1 | 381/11716 | 3.00 | 1.00 |
| Q2 | 444/11076 | 3.71 | 1.18(1.03,1.37) |
| Q3 | 491/10394 | 4.38 | 1.34(1.16,1.55) |
| Q4 | 487/9529 | 4.75 | 1.47(1.27,1.70) |
| P for trend |  |  | <0.0001 |
| Time exposure duration |  |  |  |
| 0 year | 935/24672 | 3.50 | 1.00 |
| 2 years | 407/8949 | 4.21 | 1.16(1.03,1.32) |
| 4 years | 260/5185 | 4.65 | 1.26(1.09,1.45) |
| 6 years | 201/3909 | 4.79 | 1.28(1.09,1.50) |
| P for trend |  |  | 0.0001 |

or lipid-lowering drugs(n=11,408)

Note: IR, incidence rate per 1000-person years; Model adjusted for age, sex, BMI, LDL-C, TC, hs-CRP, eGFR, current smoker, current drinker, physical activity, education level, hypertension, diabetes mellitus.

.

**Table S5** Sensitivity analysis of additionally adjusted for AIP at baseline

|  | Case/Total | IR | HR(95%CI) |
| --- | --- | --- | --- |
| Quartiles |  |  |  |
| Q1 | 513/13530 | 3.50 | 1.00 |
| Q2 | 638/13531 | 4.38 | 1.16(1.02,1.31) |
| Q3 | 752/13531 | 5.17 | 1.33(1.16,1.52) |
| Q4 | 839/13531 | 5.80 | 1.44(1.23,1.70) |
| P for trend |  |  | <0.0001 |
| Time exposure duration |  |  |  |
| 0 year | 1297/29549 | 4.07 | 1.00 |
| 2 years | 630/11613 | 5.04 | 1.15(1.03,1.28) |
| 4 years | 442/7222 | 5.71 | 1.24(1.08,1.42) |
| 6 years | 373/5739 | 6.09 | 1.25(1.06,1.47) |
| P for trend |  |  | 0.0031 |

Note: IR, incidence rate per 1000-person years; Model adjusted for age, sex, BMI, LDL-C, TC, hs-CRP, eGFR, current smoker, current drinker, physical activity, education level, hypertension, diabetes mellitus, hypoglycemic drugs, anti-hypertensive drugs, lipid-lowering drugs and Aip index at baseline.

**Table S6** Sensitivity analysis of using another method to calculate the cumulative AIP

|  | Case/Total | Incidence rate, per 1000 person-years | Model 1 | Model 2 | Model 3 |
| --- | --- | --- | --- | --- | --- |
| Quartiles |  |  |  |  |  |
| Q1 | 525/13530 | 3.60 | 1.00 | 1.00 | 1.00 |
| Q2 | 624/13531 | 4.27 | 1.23(1.09,1.38) | 1.14(1.01,1.29) | 1.14(1.01,1.28) |
| Q3 | 747/13531 | 5.12 | 1.49(1.34,1.67) | 1.34(1.19,1.51) | 1.33(1.18,1.50) |
| Q4 | 846/13531 | 5.85 | 1.73(1.55,1.94) | 1.46(1.30,1.65) | 1.44(1.28,1.62) |
| P for trend |  |  | <0.0001 | <0.0001 | <0.0001 |
| Time exposure duration | | |  |  |  |
| 0 year | 1345/30458 | 4.09 | 1.00 | 1.00 | 1.00 |
| 2 years | 639/11467 | 5.18 | 1.31(1.20,1.45) | 1.20(1.09,1.33) | 1.19(1.08,1.32) |
| 4 years | 414/6868 | 5.62 | 1.43(1.29,1.60) | 1.27(1.13,1.42) | 1.25(1.11,1.40) |
| 6 years | 344/5330 | 6.04 | 1.56(1.39,1.76) | 1.31(1.15,1.49) | 1.29(1.14,1.47) |
| P for trend |  |  | <0.0001 | <0.0001 | <0.0001 |

Note: IR, incidence rate per 1000-person years; Model adjusted for age, sex, BMI, LDL-C, TC, hs-CRP, eGFR, current smoker, current drinker, physical activity, education level, hypertension, diabetes mellitus, hypoglycemic drugs, anti-hypertensive drugs, lipid-lowering drugs. Another method for calculating cumulative AIP: cumAIP=[(AIP2006 + AIP2008)/2 × time 2006–2008 + (AIP2008 + AIP2010)/2 × time2008–2010)].

**Table S7** Reclassifcation and discrimination statistics for changes in cumulative AIP

|  | C statistics | | IDI | | Continuous NRI | |
| --- | --- | --- | --- | --- | --- | --- |
|  | Estimate (95% CI) | *P* value | Estimate (95% CI), % | *P* value | Estimate (95% CI), % | *P* value |
| Ischemic stroke |  |  |  |  |  |  |
| Original model | 0.7058(0.6970,0.7147) |  | Reference |  | Reference |  |
| Original model + Aip_2006_ | 0.7061(0.6972,0.7150) | 0.0009 | 0.000152(0,0.0003) | 0.0038 | 0.1106(0.0713,0.1498) | <0.0001 |
| Original model + Aip_2010_ | 0.7074(0.6985,0.7163) | <0.0001 | 0.000358(0.0002,0.0005) | 0.0001 | 0.1279(0.0887,0.1670) | <0.0001 |
| Original model + cumAIP | 0.7102(0.7014,0.7190) | <0.0001 | 0.000411(0.0002,0.0006) | <0.0001 | 0.1346(0.0954,0.1738) | <0.0001 |

NRI net reclassification index, IDI integrated discrimination improvement, AIP Atherogenic index of plasma

The Original model is the China-par model.
